# A Comparison of Methamphetamine-Induced Psychosis and Schizophrenia: A Review of Positive, Negative, and Cognitive Symptomatology

**DOI:** 10.3389/fpsyt.2018.00491

**Published:** 2018-10-10

**Authors:** Travis A. Wearne, Jennifer L. Cornish

**Affiliations:** ^1^Department of Psychology, Macquarie University, Sydney, NSW, Australia; ^2^School of Psychology, University of New South Wales, Sydney, NSW, Australia

**Keywords:** methamphetamine, psychosis, schizophrenia, positive symptoms, negative symptoms, cognition

## Abstract

Methamphetamine is a potent psychostimulant that can induce psychosis among recreational and chronic users, with some users developing a persistent psychotic syndrome that shows similarities to schizophrenia. This review provides a comprehensive critique of research that has directly compared schizophrenia with acute and chronic METH psychosis, with particular focus on psychiatric and neurocognitive symptomatology. We conclude that while there is considerable overlap in the behavioral and cognitive symptoms between METH psychosis and schizophrenia, there appears to be some evidence that suggests there are divergent aspects to each condition, particularly with acute METH psychosis. Schizophrenia appears to be associated with pronounced thought disorder, negative symptoms more generally and cognitive deficits mediated by the parietal cortex, such as difficulties with selective visual attention, while visual and tactile hallucinations appear to be more prevalent in acute METH-induced psychosis. As such, acute METH psychosis may represent a distinct psychotic disorder to schizophrenia and could be clinically distinguished from a primary psychotic disorder based on the aforementioned behavioral and cognitive sequelae. Preliminary evidence, on the other hand, suggests that chronic METH psychosis may be clinically similar to that of primary psychotic disorders, particularly with respect to positive and cognitive symptomatology, although negative symptoms appear to be more pronounced in schizophrenia. Limitations of the literature and avenues for future research are also discussed.

## Methamphetamine

Amphetamines refer to a class of chemically related compounds that have been used extensively over the last century in both recreational and medicinal settings, with various amphetamine analogs used in the treatment of narcolepsy, attention deficit hyperactivity disorder (ADHD) and obesity (1, 2). Methamphetamine (METH; N-methyl-alpha-methylphenethylamine) is a highly potent amphetamine derivative that is frequently abused worldwide and has significant effects on physical, behavioral, cognitive and psychiatric output (3). It is a cationic molecule and chiral compound based around a phenylethylamine core (4), and distinguishable from its amphetamine analogs by an additional methyl group. This methyl addition reportedly makes METH highly lipophilic, thereby allowing it to increasingly penetrate the blood-brain barrier (5). This causes changes to dopaminergic, serotonergic, and noradrenergic systems via the stimulated release of monoamines, the inhibition and reversal of monoamine reuptake, inactivation of presynaptic vesicular monoamine transporter 2, and by reducing the efficacy of monoamine metabolic enzymes (6–10). Although all three monoamine systems are involved, the behavioral and reinforcing properties of METH have typically been associated with dopaminergic neurotransmission, particularly in the mesocorticolimbic pathway (11, 12).

Epidemiological studies place amphetamine-type stimulants as the most widely used illicit drug in the world after cannabis (13, 14), with up to 51 million users globally between 15 and 64 years old (15–17). Worldwide statistics on METH use describe it as a global phenomenon, with METH consumption reportedly independent of wealth, geographical location, and culture (18). Recent reports suggest an increased production of METH around the world and an increasing popularity of METH over the last 5–15 years, which has been linked to increased ease and cost-effective synthesis in clandestine laboratories and augmented importation of METH from Mexico and Asia (16, 19). Indeed, worldwide seizures relating to METH have been greater than any other drug category (17). Additionally, admissions to treatment programs for METH use increased 255% from 1997 to 2007 in the USA (20, 21), although there is some evidence that the rate of admissions for METH in the USA have remained stable or slightly declined from 2004 to 2014 (22). In Australia, there has been a 233% increase in demand for METH related treatment and a 274% increase in METH related hospital admissions since 2010 (23, 24), with Queensland specifically witnessing a 20-fold increase in METH related hospital admissions from 2009 to 2015 (25).

METH is available in various forms and at different levels of chemical purity. When injected, snorted, or inhaled, METH has direct access to the circulatory system and therefore has more immediate effects on the brain (26, 27). Given that the negative consequences of METH are associated with the use of more potent forms of the drug and with hazardous routes of administration (i.e., injection), the increased availability of crystalline METH on the illegal market had resulted in a significant increase in METH's popularity amongst dependent and intravenous drug-taking populations (28). Indeed, while 22% of METH users reported that crystallized METH was their drug of choice in 2010 (29), this had increased to 50% by 2013 (30). These trends are salient given the potential for addiction and overdose with more potent forms of METH administration.

## Acute methamphetamine psychosis

Dependency to METH, together with high doses and recreational METH use, have all been associated with the induction of psychotic symptoms, including auditory and visual hallucinations, persecutory delusions, ideas of reference, and disorganized speech (31–33). The idea that METH use could induce a psychotic state has long been recognized by clinicians in Japan, who increasingly observed psychosis in their METH-dependent patients (34). The early identification of this relationship was due, in part, to the high prevalence of METH use together with the absence of polydrug use, thereby enabling clinicians to isolate the link between METH and psychosis without the confound of additional substance use (35).

Research has shown that METH psychosis is a prevalent health concern among recreational users. Studies on prevalence rates have varied between 7% (32) up to 76% (36), with a recent meta-analysis indicating that the prevalence of METH-induced psychotic disorder was 36.5% (37) and these rates were higher for lifetime prevalence (42.7%) and for those with METH use disorder (43.3%). In an Australian study of non-treatment seeking METH users, 13% of the sample population were positive for psychosis at the time of assessment (32) while 23% reported ‘clinically significant’ symptoms of psychosis over the previous year. Another study found that 60% of METH-dependent individuals sampled in the USA reported at least one type of psychotic symptom (38). Overall, recreational METH users are two to three times more likely to experience psychotic symptoms than the general population (39), with their risk increasing if they began using METH at a younger age or if large amounts of METH are administered (40). Regular METH users, on the other hand, are 11 times more likely to experience psychosis than the general population (41), with the average time between first use and onset of psychosis being 1.7 years (42). Furthermore, users of crystallized METH are more likely to report psychotic symptoms compared to other forms of METH (43), suggesting that the type and route of administration may be important factors in determining the likelihood of psychotic symptoms.

Users are more susceptible to the psychotic effects of METH whilst they are using the drug. McKetin et al. (15) found that chronic METH users were 5 times more likely to experience psychotic symptoms during periods of METH use than during periods of abstinence. They also found a dose-response effect between the frequency of METH use and psychotic symptoms, with psychosis reaching a peak likelihood of 48% following 16 days or more of chronic use. Importantly, these findings were still significant after controlling for polydrug use, suggesting that the psychotic symptoms were attributable to the effects of METH and not due to the interaction of additional drug consumption. Overall, these findings suggest that METH use is associated with a high prevalence of psychotic symptoms, which may present a significant burden on the healthcare system due to increased demand for care and management of METH-related psychoses. Indeed, METH psychosis accounted for 10% of admissions to psychiatric facilities in Thailand (44), and in Australia, METH psychosis was responsible for 10.3 hospital admissions per 1,000 (45). More recent data has also suggested an increase in METH psychosis admissions to hospital emergency rooms and psychiatric facilities over recent years. For example, the number of admissions to psychiatric units for METH psychosis in Queensland has increased significantly from 2012 (25) while in New South Wales, Australia, the number of hospital admissions for METH psychosis declined in the mid-2000s but have steadily increased again since 2010 (46). These findings also appear to be independent of geographical location, with increased emergency department admissions for METH psychosis reported in the Americas (47) and the middle east (48).

## Chronic methamphetamine psychosis

METH psychosis typically follows a transient course, with symptoms subsiding once the user has stopped taking the drug (3). Some consumers, however, can experience a prolonged psychosis that persists even after the drug has cleared from the body, with the majority of psychotic symptoms resolving within 1 month (34, 49). Some research has further indicated that METH psychosis can develop into an enduring form of psychosis. Reports have suggested that up to 30% of those with METH psychosis may have symptoms that continue up to 6 months following abstinence (49), with specific studies reporting 15–28% of patients admitted to hospital with METH psychosis needing hospitalization for more than 2–3 months following admission (50, 51). Additionally, others have reported that 10–28% of patients with METH psychosis continued to display psychosis for more than 6 months (35, 50), while in another study, 28% of METH-users continued to display “schizophrenia-like symptoms” 8–12 years following abstinence (52). Outside of Japan, McKetin et al. (15) reported that even abstinent METH users had a 7% risk of experiencing psychotic symptoms and another group found that 5% of abstinent METH-dependent users met criteria for a psychotic disorder at 3 years follow-up (53). Furthermore, METH can induce a chronic psychosis in those with no premorbid psychiatric risk factors (54), suggesting that METH use can induce persistent physiological changes consistent with psychosis that are independent to genetic and personality predispositions. It is recommended that readers examine many of the comprehensive review articles available for further information on the clinical profiles, correlates, and recovery of METH-induced psychosis (55–60). Overall, METH psychosis can result in a persistent psychotic syndrome that is resistant to spontaneous recovery, and in light of the high use of METH use globally, chronic METH psychosis will undoubtedly continue to be an issue for health-care professionals. As such, understanding the factors that subserve the neurobiology and maintenance of chronic psychosis induced by METH abuse will be important for delineating diagnostic markers and avenues for treatment.

## Schizophrenia

Schizophrenia is a severe, complex and debilitating neuropsychiatric disorder that is traditionally associated with poor treatment outcomes relative to other psychiatric disorders. It is a significantly heterogeneous disorder, with symptoms so diverse and idiosyncratic from patient to patient that the clinical profile has to be “clustered” into different domains. While there is no symptom that is sufficient for a person to be diagnosed with schizophrenia, there are particular symptoms that aid in differential diagnosis. “Positive symptoms” refer to symptoms that are usually not present but are experienced by those with schizophrenia, and include distortions in perceptions (hallucinations), false beliefs or distorted thought content (delusions), unclear or confused thinking (thought disorder), and disorganized speech. These symptoms are generally interpreted as a loss of touch with reality and are present at discrete times during “psychotic episodes,” which are considered a core feature of the disorder (61). While these symptoms can also be present during remission, medication serves to suppress the severity and chronicity of these symptoms. “Negative symptoms,” on the other hand, refer to symptoms or experiences that are usually absent or diminished in individuals with schizophrenia. These include social withdrawal, anhedonia, flattened affect, motor retardation, and poverty of speech (62, 63). Negative symptoms have a significant bearing on functional engagement and independence, with negative symptoms shown to predict the status of future functioning, employment, independence, and social contact (64).

While both positive and negative symptoms are established as core symptom dimensions and criteria for schizophrenia diagnosis in the DSM-5 (65), a third core domain reported is cognitive dysfunction. A wide range of cognitive domains appear to be compromised in schizophrenia, with many reviews and meta-analyses concluding moderate to severe deficits in general intelligence, attention, working memory, verbal learning and memory, speed of information processing, visuospatial deficits, and executive dysfunction (66–71). The cognitive deficits in schizophrenia are stable across the course of the disorder (72–75) and are consistent between those with first episode psychosis and chronic schizophrenia (76–78). However, there is some evidence that those with earlier onset schizophrenia may have a decline in cognitive function throughout the progression of the illness (79). Furthermore, antipsychotic medication appears to have minimal positive impact, if at all, on the cognitive difficulties associated with schizophrenia (80). Executive function appears to be the most compromised and conserved cognitive deficit across patients with schizophrenia (81, 82), with executive deficits shown to be the most pervasive amongst older adults with schizophrenia (83) and negatively impacted by number of psychotic episodes (84). Additionally, the fact that the cognitive issues in schizophrenia are deleterious to social functioning, functional outcomes (85, 86), independence (87, 88), recovery (89), and well-being (90) has prompted the argument that cognitive dysfunction should be regarded as one of the core dimensions in the disease, particularly with respect to DSM-5 diagnostic criteria (61).

## The relationship between METH-induced psychosis and schizophrenia

While a subset of METH users can experience an enduring form of psychosis, there is uncertainty of the diagnostic status of chronic METH psychosis as a primary psychotic disorder. That is, METH-induced and other substance-induced psychoses are clearly distinguished from schizophrenia and other primary psychoses in the Diagnostic and Statistical Manual. In fact, any psychosis during the withdrawal from a substance requires the diagnosis of “substance-induced psychotic disorder” according to the Diagnostic and Statistical Manual (65) and the International Classification of Diseases, Tenth Revision (ICD-10). Diagnostic guidelines, however, become ambiguous should the psychosis persist for an extended period of time. The DSM-5 outlines that any psychosis that persist longer than 6 months should warrant the diagnosis of a primary psychotic illness (65). Indeed, a Thai study of METH abusers, who were initially hospitalized for METH psychosis found that 38.8% had been diagnosed with schizophrenia due to persistent psychosis at 5 years follow up (91), and 5.0% of Chinese patients with METH-induced psychosis had their diagnosis changed to schizophrenia (49). Longitudinal analyses have also found that 19.1% (92) to 30% (93) of patients initially admitted for amphetamine-induced psychosis had transitioned to a schizophrenia diagnosis at follow-up. Furthermore, a large study conducted over a 10 year period in the USA determined that individuals who were hospitalized for METH-related causes had a higher risk of receiving a subsequent schizophrenia diagnosis (94). Therefore, while the potential for METH to induce an acute psychosis is well recognized, the development of an enduring-form of psychotic disorder, and its potential to transition into a primary psychotic disorder, such as schizophrenia, is not as well understood. As these studies propose that stimulant-induced psychoses represents a significant precursor to the development of more enduring forms of psychotic disorders, these findings should guide the management, early intervention and policy related to METH-related psychoses to circumvent the progression of these conditions.

While the above findings support that METH use is associated with an enduring psychosis, there are several interpretations of the link between METH use and schizophrenia. Firstly, METH could induce schizophrenia by eliciting an underlying vulnerability/predisposition to a primary psychotic disorder. Early research on amphetamine psychosis attributed the continuation of psychotic symptoms to “latent paranoia” (95). Additionally, a growing body of literature has examined the role of genetic and environmental interactions in the development of METH psychosis, with some studies showing convergence of genetic risk factors for METH psychosis with those for schizophrenia (58, 96). Additionally, one study found a significant enrichment of singe nucleotide polymorphisms (SNPs) for METH psychosis risk in patients with schizophrenia (97) while another found that a family history of schizophrenia was a risk factor for the development of METH psychosis (40, 42, 98). These findings suggest that the development of a persistent psychotic syndrome, such as schizophrenia, may be the complex interaction between a predetermined vulnerability and/or the direct effects of METH as an environmental trigger (i.e., the two hit hypothesis), and may provide an explanation as to why only a small percentage of those with METH psychosis go on to develop a persistent psychotic syndrome. More recently, however, there has been discussion surrounding the possibility that METH use could actually *cause* the onset of schizophrenia (54, 94, 99), potentially by inducing schizophrenia pathology. Even though this does not explain why only a percentage of users develop a persistent psychotic syndrome, both explanations suggest that METH psychosis and schizophrenia may be the same disorder on a continuum of pathology, converging with the idea that schizophrenia is a neurobiological disorder with multiple etiologies.

Alternative explanations for METH-induced psychosis may be possible. As such, it could be that METH psychosis and schizophrenia represent distinct disorders, and indeed, several researchers have proposed that METH use in isolation can produce a persistent psychotic syndrome that should be diagnosed and treated as a distinct syndrome to schizophrenia (100, 101). Therefore, given that any persistent psychosis beyond a 6-month period should be considered as a primary psychotic disorder, based on the current diagnostic criteria in the DSM-5, METH psychosis may be routinely misdiagnosed and treated as schizophrenia. Therefore, the diagnosis of schizophrenia secondary to METH use described in the aforementioned studies may merely reflect adherence to diagnostic protocol and may not be a true reflection of the status and prevalence of chronic METH psychosis in the general population. That is, individuals who present with METH psychosis may be diagnosed with schizophrenia, which may therefore underestimate the degree to which METH use results in a persistent psychotic disorder in epidemiological research studies.

Overall, there appears to be uncertainty about whether METH use causes schizophrenia or whether chronic METH psychosis represents a symptomatically distinct disorder that should be distinguished from other primary psychoses. While there appears to be similarity between the two conditions, there is limited research that has explicitly compared the behavioral and cognitive markers between the disorders. To understand the similarities and distinguishing features of METH psychosis and schizophrenia is of benefit. Not only will this assist in determining the diagnostic entity of METH psychosis, but will also help develop differential diagnostic markers for clinicians, better treatment options for long-term METH psychosis suffers, and will help to delineate common biological markers across syndromes that may initiate and maintain a persistent vulnerability to psychosis. This will enable a deeper theoretical understanding of the specific biological factors that subserve the symptoms that are commonly observed across psychotic disorders.

## Overview of review

### Aims

The current review will describe and critique the literature that has compared the clinical profile of schizophrenia with (i) acute METH psychosis and (ii) chronic METH psychosis, with particular focus on positive, negative and cognitive symptoms. While several reviews have examined the clinical profiles, risk factors, and correlates of METH-psychosis (55, 59, 60) and cognitive deficits associated with METH use (102–104), the aim of this study was to provide a comprehensive overview of research that has directly compared METH psychosis (acute and chronic) with schizophrenia. Furthermore, while (55) have reviewed the relationship between METH psychosis and schizophrenia, we wished to extend this review by differentiating between acute and persistent forms of METH psychosis. If the use of METH does cause a primary psychotic disorder, then the presentation and symptoms of chronic METH psychosis should match those typically reported in schizophrenia. Consequently, persistent METH psychosis could be regarded as the same diagnostic entity and could allude to similar neurobiology and etiological mechanisms. However, if METH psychosis represents a biologically and clinically distinct disorder there should be divergence in the behavioral, cognitive and biological markers between METH psychosis and schizophrenia.

### Inclusion criteria

Prior to conducting the literature search, inclusion criteria were formulated from the aims, not only to determine which studies would be suitable but to provide a unique perspective to the review and to also minimize the occurrence of methodological flaws. These included: (1) studies had to based on people, aged 16 years and older; (2) the current review focused specifically on research relating to methamphetamine (rather than amphetamine or other psychostimulants); (3) studies had examined profiles associated with METH psychosis (i.e., no studies looking at the cognitive effects of METH without psychosis); (3) studies had to have directly compared METH psychosis with schizophrenia or primary psychotic disorder (4) METH usage had to precede the presentation of psychosis in order to focus on METH-induced psychotic syndromes; (5) only original research studies were included (i.e., reviews were omitted); (6) Case studies were omitted [for a review and examination of historical case studies of METH psychosis, please see (59)].

### Search approach

To identify potential studies for inclusion in this review, the computerized databases of PubMed, PsychINFO and ScienceDirect were searched. Additionally, reference lists from retrieved articles were screened to identify omitted articles from the database search. Lastly, a Google Scholar search was conducted to ensure that no main article escaped detection in the literature search. The following search terms were used to identify potential articles: (methamphetamine psychosis OR methamphetamine-induced psychosis) AND (schizophrenia OR primary psychotic disorder) AND (negative symptoms OR positive symptoms OR psychiatric symptoms OR cognition).

## Positive symptoms

An overview of the design and findings of individual research studies that have directly compared METH psychosis with schizophrenia can be found in Table 1. The methodological considerations of this research are detailed in Table 2.

**Table 1:** Summary of experimental studies that have compared METH psychosis with schizophrenia.

**Study**	**Design**	***n***	**Sample characteristics**	**Groups**	**Psychosis type**	**Measures used**	**Findings**
(105)	Cross sectional, case-control study	252	Sample consisted of 160 METH users and 54 patients with schizophrenia. They were recruited from various detention centers, hospitals, psychiatric facilities and in- and outpatient clinics in Taiwan. The control group were recruited from community volunteers.	5 groups: Control group (*n* = 67), METH users (*n* = 25), Acute MAP (*n* = 50), Persistent MAP (*n* = 56) and Schizophrenia (*n* = 54).	Both	Diagnostic Interview for Genetic Studies (Chinese Version; DIGS-C, BPRS and BACS	*BPRS Total:* Schiz = Persistent MAP, Both Schiz and Persistent MAP > Acute MAP. *BPRS Positive Symptom*s: Schiz = Persistent MAP, Both Schiz and Persistent MAP > Acute MAP.*BPRS Negative Symptoms*: Schiz > Persistent MAP > Acute MAP. *BACS*: Schiz = Persistent MAP across all cognitive measures. Schiz +Persistent MAP < Acute MAP +METH users across all cognitive measures. No differences between Acute MAP, METH users and Controls across all cognitive measures.
(106)	Cross sectional study between MAP and Schiz	90	Clinical participants were recruited from the emergency ward of the Iran Psychiatric Hospital and enrolled into the study after stabilization. Diagnoses were obtained from patient files.	3 groups: MAP (*n* = 30), schizophrenia (*n* = 30) and healthy controls (*n* = 30)	Acute MAP	Wisconsin Card Sorting Test (WCST), Stroop Test, Visual Search and Attention Test (VSAT) and Wechsler Memory Scale (WMS)	MAP + Schizophrenia < Controls on WCST, Stroop, VSAT and WMS. No sig differences between MAP And Schizophrenia for WCST, Stroop and WMS. Schiz performed worse than MAP on VSAT.
(107)	Cross-sectional study as part of 12-month prospective study.	198	Current METH users (61% male). Average age of 31.65 years. METH was primary drug of choice. Recruited via needle syringe programs in Australia	Psychotic (51%) and non-psychotic disorder groups (49%). Psychotic disorder separated into lifetime (39%) and current diagnoses (61%) and subdivided into those with substance-induced and those with primary psychotic disorders	Acute MAP	BPRS	No sig differences between substance-induced psychosis and primary psychotic disorder on total BPRS scores, positive symptoms, negative symptoms, mania, and depression-anxiety.
(108)	Cross sectional study between MAP and Schiz	39	Chart review was used to select participants for the study. Schizophrenia diagnosis was confirmed by hospital records.	MAP (*n* = 19) and paranoid schizophrenia (*n* = 20)	Likely acute MAP, although abstinence or time since admission was not reported	Wechsler Abbreviated Scale of Intelligence (WASI), Repeatable Battery for the Assessment of Neuropsychological Status (RBANS), Delis Kaplan Executive Functioning System (DKEFS) Color-word Interference Test, Continuous Performance Test of Attention, Grooved Pebgoard, Wide Reading Achievement Test (WRAT) reading subtest and Trailmaking Tests (TMT)	No significant differences were observed between groups on any cognitive domain examined.
(109)	Cross-sectional study as part of larger longitudinal study	284	Derived from a later study, the Methamphetamine Treatment Evaluation Study (MATES). Participants had a mean age of 31.6 years and 71% male.	4 groups: METH users (*n* = 110), Acute MAP (*n* = 85), Persistent MAP (*n* = 37), Primary Psychosis (*n* = 52)	Both	BPRS and CIDI	Transient MAP > Control group on lifetime persecutory delusions and tactile hallucinations. Persistent MAP > Transient MAP on lifetime delusions of reference, thought interference, complex auditory hallucinations and hallucinations in various modalities (visual, olfactory and tactile). Primary psychosis > Transient MAP on delusions of reference, thought projection, erotomania, passivity, and auditory, olfactory and tactile hallucinations. No sig difference between persistent MAP and primary psychoses on any positive symptom.
(110)	Cross-sectional study between MAP and Schiz	285	Participants were admitted to two psychiatric wards in public hospitals in Norway. 52% were men and average age was approximately 38-39 years.	METH negative diagnosed with schizophrenia (*n* = 33) vs. METH positive with psychosis (*n* = 9)	Acute MAP	Urine and/or blood analysis to confirm the presence of METH. PANSS	No sig difference between MAP and Schizophrenia on any positive symptom
(111)	Experimental study between MAP and schizophrenia	56	Participants were recruited from the University of Tokyo Hospital and Tokyo Metropolitan Matsuzawa Hospital.	3 groups: MAP (*n* = 21), schizophrenia (*n* = 14) and healthy controls (*n* = 21)	Acute MAP	GAF, PANSS, JART, Stop-signal Task and NIRS	No sig difference between MAP and Schizophrenia groups on the Positive and Negative subscales of the PANSS. Using the PANS 5-factor model, MAP group had higher Excitement scores compared to schiz. Trend (*p* = 0.052) for Schiz to have lowest percent correct on stop-signal task compared to MAP and controls. Both MAP and Schiz showed reduced activation in the ventrolateral prefrontal cortex compared to controls. MAP had reduced activation in the frontopolar prefrontal cortex compared to schizophrenia
(112)	Cross sectional study between MAP and Schiz	102	Data was collected from two larger cross-sectional studies. Data was collected from patients presents with psychotic disorders admitted to a psychiatric facility in Cape Town.	MAP (*n* = 33) and schizophrenia (*n* = 69)	Acute METH Psychosis	SCID-I-RV (Structured Clinical Interview for DSM-IV)	Thought broadcasting was more prevalent in Schizophrenia (42%) than in MAP (24%) and significantly predicted the diagnosis of schizophrenia once controlling for age. Auditory hallucinations (voices heard conversing) were significantly higher in MAP (48.5%) than in schizophrenia (20.3%). No difference in the severity and prevalence of any other first-rank symptoms between MAP and Schiz.
(113)	Cross-sectional study on retrospective data	61	Data for both groups was taken from the WHO-MAIP and RLAI-Thai studies. The MAP group had used METH for and average of 3.8 (5.4) years.	MAP group (*n* = 168) and schizophrenia (*n* = 169)	Acute MAP	Mini-International neuropsychiatric Interview- Plus (MINI-P), Manchester Scale	No significant differences between MAP and schiz on the severity of negative symptoms. MAP group had higher positive symptoms scores of delusions, hallucinations and incoherent speech. Differential item functioning analysis further showed that MAP and Schiz were able to be differentiated based on incoherent speech alone. The positive and negative symptom profiles of MAP and Schiz were the same.
(114)	Cross sectional study between MAP and Schiz	22	Chronic MAP group had used moderate and/or high doses of MAP intravenously for an extended period of time. The MAP group were recruited from inpatient and outpatient settings in Japan. The Schiz group were matched to the MAP group and recruited from the same hospital	MAP group (*n* = 11) and paranoid schizophrenia (*n* = 11)	Chronic MAP	Scale for the Assessment of Negative Symptoms (SANS). Medical records were examined for positive symptom profiles on admission.	Qualitatively, the positive symptom profile was similar between both MAP and Schiz. Overall negative symptoms were milder in MAP compared to Schiz. Affective flattening or blunting and alogia were less severe in the MAP group compared to Schiz.
(115)	Cross sectional and case-control study between MAP and Schiz	106	Sample consisted of METH users with psychosis and patients with schizophrenia. They were recruited from various general hospitals, psychiatric facilities and in- and outpatient clinics in Taiwan.	MAP (*n* = 53) and schizophrenia (*n* = 53)	Chronic MAP	BPRS, PANSS and structure interview questions in the DIGS-C	No difference in the patterns of delusions experienced between MAP and Schiz. Auditory hallucinations were comparable between both MAP and Schizophrenia. Visual and Tactile hallucinations were more prevalent in MAP compared to schizophrenia. Unusual thought content, blunted affect, emotional withdrawal and motor retardation were more prevalent in Schiz than persistent MAP. Schiz was associated with greater negative symptoms overall than MAP
(116)	Experimental study between MAP and schizophrenia	34	Recruited through in- and outpatient clinics in Japan.	MAP (*n* = 15) and Schizophrenia (*n* = 19)	Chronic MAP	Japanese version of the National Adult Reading Test (JART), PANNS, Brief Assessment of Cognition in Schizophrenia (BACS), verbal fluency task, NIRS measurements	No differences between groups on PANNS total score, positive symptoms and negative symptoms. No differences between groups on tasks of verbal memory, working memory, motor speed, verbal fluency, attention and processing speed, executive functioning, and total cognition score. Oxyhaemoglobin changes in the prefrontal cortex were higher in MAP compared to schizophrenia, particularly in the right dorsolateral prefrontal cortex

**Table 2:** Methodological assessment of studies that have compared METH psychosis with schizophrenia.

**Study**	**Methodological considerations**						
	**Schizophrenia comparison group?**	**Healthy control group?**	**Differentiate between acute and chronic psychosis?**	**METH psychosis characteristics**	**Abstinent at time of assessment?**	**Other factors controlled for?**	**Other considerations?**
(105)	Yes	Yes	Yes	Acute group was defined as METH users with brief psychotic symptoms that disappeared 1 month after ending METH. Those who continued to experience psychosis 1 month after abstinence from METH were categorized as METH users with persistent psychosis.	Yes	Those in the METH groups could not have a history of psychosis prior to drug use and the psychosis had to be clearly linked to drug use. Schizophrenia participants could not have a history of drug or alcohol use disorders.	Those in the acute METH psychosis were assessed approximately 9 weeks after experiencing their METH psychosis so could not be assessing their psychotic symptoms and cognitive functioning in the immediate time following their episodes. The control group was also not perfectly matched to the METH users. Neuroleptic medication also differed between groups
(106)	Yes	Yes	No	MAP group were recruited from emergency room so experiencing psychosis at the time. Could represent those with acute MAP.	Not stated, although recruited via emergency room so likely to have been active METH users at time of enrolment.	Matched on age, gender and education. Those in Schiz group had no history of METH use.	66% of MAP group had history of other drug use. Those in the MAP and Schiz group were taking neuroleptic medication. Small sample sizes
(117)	Yes (NFP group)	No	No	Psychotic symptoms followed recent METH use or cessation of prolonged and heavy METH use	Not stated. Unsure if sample was abstinent after discharge too.	No differences in age, gender or education. Polydrug use not addressed.	NFP group was a heterogeneous group consisting of a group of schizophrenia (*n* = 46), schizoaffective disorder (*n* = 1) and brief psychotic disorder (*n* = 3). 9 patients in the NFP had a history of METH use.
(107)	Yes	No	No.	Differentiates between lifetime and current psychotic disorder but not acute and chronic METH psychosis	Yes	No sig differences between groups on any demographic variable examined. No differences between groups on polydrug use	Primary psychotic disorder group were also METH users and may not represent pure schizophrenia.
(108)	Yes	No	No	Had to meet diagnostic criteria for METH dependence concurrently with psychotic disorder and the dependence had to precede the psychosis.	Not stated	METH use had to precede the onset of psychosis for the MAP group. No differences were found in sex, education, legal status, and medication.	Differences were found between groups in age, ethnicity and place of birth. Small sample sizes.
(109)	Yes	No	Yes	Acute METH defined as participants who experience psychosis symptoms when using METH for at least 1 months but no during months when abstinent. Persistent MAP was defined as experiencing psychotic symptoms during METH use and for at least 1 months or longer after abstinence	Not stated		Details *lifetime* positive symptoms rather than positive symptoms at the time of the assessment. Primary psychotic disorder group were also METH users and may not represent pure schizophrenia.
(110)	Yes	No	No	Had to test positive for METH in system to be included in the study.	13% had taken methamphetamine recently according to blood and/or urine analysis	Those in the schizophrenia group did not test positive for METH	35 of 38 tested positive for METH and Amphetamine. 87% also had at least one other psychoactive substance in their urine and/or blood. Polydrug use may be a confounding factor. Small sample size of those who are METH positive with psychosis.
(111)	Yes	Yes	No	6 MAP subjects were classified with 'Psychotic disorder due to use of METH' and the remaining 15 were classified with 'residual and late-onset psychotic disorder due to use of METH'	Not stated	Groups were matched for age and gender. Medication dosage no different between MAP and Schiz.	IQ > in healthy control group. No analyses were done to look at the distinction between acute and persistent MAP due to small sample size.
(112)	Yes	No	No	Symptom onset had to be within 1 months of METH intoxication or withdrawal and could not exceed 4 weeks.	No greater than 4 weeks	Schiz group had exclusion criteria of previous substance use while MAP were excluded if meeting dependence criteria for any substance other than METH. No difference between groups on education and gender distribution	Thought broadcasting was significantly only once the age of the samples were controlled.
(113)	Yes	No	No	MAP group taken from hospitals and had to have used METH in the previous week. Could represent both acute and chronic MAP individuals.	Had to have used METH in the past week	Not matched for age. Unsure of drug taking habits of those in the Schiz group	The schiz group had all been taking neuroleptic medication for months and/or years while the MAP group had only recently comment antipsychotic medication. Those in the schiz group were chosen for the study as they were not responding well to medication and may not truely represent schizophrenia.
(114)	Yes	No	No	Chronic psychosis. Subjects had to have continued to experience delusions and hallucinations for more than 1 month after abstinence from the drug.	Not stated	Matched for age and gender.	64% of sample had a history of drug dependency besides METH. Positive symptoms were documented qualitatively from the medical records from admission. Small sample size.
(115)	Yes	No	Yes	Individuals had to have an enduring psychosis for more than 1 month after cessatin of METH	Greater than 1 months	Those in the METH groups could not have a history of psychosis prior to drug use and the psychosis had to be clearly linked to drug use. Schizophrenia participants could not have a history of drug or alcohol use disorders.	
(116)	Yes	No	No	8.42 years since onset of psychotic symptoms may suggest the sample was more chronic MAP than acute. No evidence of abstinence so assumed they are acute MAP	Not stated	No differences in age, gender, medication and premorbid IQ.	Nearly all subjects were on neuroleptic medication. Trend for schizophrenia group to have longer duration of illness. No indication of acute or chronic. Small sample size.

### Acute meth psychosis vs. schizophrenia

Early findings on METH induced psychosis reported hallucinations and delusions as a predominant presenting factor (34, 118), with later findings acknowledging that the similarities between METH psychosis and schizophrenia were largely directed toward positive symptoms. McKetin et al. (32) found that unusual thoughts, hallucinations and suspiciousness were present in one-quarter of chronic consumers of METH diagnosed with acute METH psychosis. Indeed, Bousman et al. (119) examined the variation in positive symptoms across individuals with METH psychosis. While they found three distinct sub-profiles, delusions were common amongst all individuals with METH-induced psychosis. Additional studies have also reported that METH psychosis is associated with a high prevalence of persecutory delusions, auditory and visual hallucinations, odd speech, and delusions of reference (15, 40, 60, 113, 119–122).

Of studies that have directly compared acute METH psychosis with schizophrenia (Table 1), researchers have found no difference in the type and severity of positive symptoms using the Positive and Negative Syndrome Scale (PANSS) (110, 117) or the Brief Psychiatric Rating Scale (BPRS) (111). There is also research demonstrating that the longitudinal changes of positive symptoms between METH psychosis and schizophrenia are similar. For example, Hajebi et al. (117) conducted a prospective study on individuals with METH-induced psychosis and found that there was no significant difference in the severity of positive symptoms (using the PANSS) between acute METH psychosis and non-affective psychosis (e.g., schizophrenia) groups on admission, at discharge, and at 6 and 12-month follow-up. These findings suggest that the progression of positive symptoms following METH psychosis is comparable to that of schizophrenia. However, given that those with METH-induced psychosis continued to experience symptoms of psychosis following discharge, it is uncertain whether this group represents acute or chronic METH psychosis. Furthermore, it should be considered that the non-affective psychosis group was a heterogeneous sample, consisting of participants diagnosed with schizophrenia, schizoaffective disorder and brief psychotic disorder. Collectively, these findings suggest that the positive symptoms of acute METH-induced psychosis are qualitatively and quantitatively comparable to the positive symptoms of schizophrenia, with the initial presentation of acute METH psychosis indistinguishable from schizophrenia-related psychosis (113).

Despite the considerable overlap in positive symptoms between acute METH psychosis and schizophrenia, there are several differences across both conditions. For example, Srisurapanont et al. (113) found that while there were no difference in the type and severity of positive symptoms between METH psychosis and schizophrenia, the METH psychosis group tended to have more severe hallucinations and delusions compared to schizophrenia. Further analysis revealed that incoherent speech, a distinguishing marker of thought disorder, was the only symptom to be differentially expressed between schizophrenia and METH psychosis. Thought disorder refers to disorganized thinking and is characterized by the loosening of associations and fragmented speech (123), and is suggested to be a defining and salient feature in schizophrenia (124–127). Although related to amphetamine, initial work by Bell (125) distinguished between schizophrenia and amphetamine-induced psychosis with the appearance of thought disorder, as this symptom was only seen in schizophrenic cases. Additionally, Yui et al. (127) found that while individuals with METH psychosis experienced paranoid hallucinations and delusions, the same participants did not exhibit thought disorder or disorganized speech. Therefore, the absence of thought disorder may be a discriminating feature associated with METH psychosis that can be used to differentiate this disorder from schizophrenia. However, the use of this potential discriminating feature this is currently based on indirect and inconclusive evidence, and further research is needed to determine the differentiation of thought disorder between METH psychoses and schizophrenia.

Studies that have also differentiated the types of hallucinations and delusions commonly experienced in METH psychosis and schizophrenia. Shelly et al. (112), in their examination of first-rank positive symptoms, found that acute METH psychosis and schizophrenia demonstrated comparable positive symptoms but those with acute METH psychosis showed higher frequency of auditory hallucinations (48.5%) in comparison to schizophrenia (20.3%). Conversely, thought broadcasting was more prevalent in the schizophrenia (42%) group compared to METH psychosis (24%), although this was only significant once age was controlled for in their analyses. Regardless, these findings are strengthened by the exclusion criteria of polydrug use for the METH psychosis group and METH use for the schizophrenia group. Also, the individuals in the METH psychosis group were deemed eligible if they were abstinent for no greater than 4 weeks, highlighting that this represented a true acute psychosis sample. There is also some evidence that persecutory delusions and tactile hallucinations may be specific to acute/transient METH psychosis as opposed to the chronic psychosis and schizophrenia (109), and indirect comparisons suggest that visual and tactile hallucinations appear to be more prominent in METH psychosis compared with schizophrenia (4, 125). Chen et al. (40) reported that 46.5 and 21.3% of their METH psychosis sample reported visual and tactile hallucinations, respectively. Additional findings have also confirmed visual hallucinations in 68.8% of METH abstinent individuals (128) while others have reported that visual hallucinations are the fourth most reported positive symptom in METH psychosis (120). However, visual hallucinations are typically only reported in severe cases in schizophrenia (129), with the prevalence rate ranging from 16 to 27% (129, 130). Additionally, formication, a tactile hallucination where individuals believe that one's skin has been infested by bugs, is typically only reported in METH psychosis (131). Therefore, while auditory hallucinations appear to be the most common hallucination of both METH psychosis and schizophrenia, visual and tactile hallucinations appear to be more prominent in METH psychosis. However, these later findings are based on indirect comparisons and not on reliable evidence that has directly compared METH psychosis with schizophrenia.

### Chronic meth psychosis vs. schizophrenia

Researchers have also examined chronic METH psychosis in relation to schizophrenia. In a small study of 11 patients with chronic METH psychosis who had been abstinent from METH for more than 1 month (114) qualitatively reported that five subjects experienced visual hallucinations, seven experienced delusions of reference and persecutory delusions while all experienced auditory hallucinations. Additionally, Yamamuro et al. (116) found similar PANSS results in their experimental study examining oxygenation changes in the prefrontal cortex in acute METH psychosis and schizophrenia during a verbal fluency task. Furthermore, Wang et al. (115) examined the positive symptom profile of 52 individuals with chronic METH psychosis (who experienced psychosis and had been abstinent from METH for more than 1 month) and compared this to 53 participants with schizophrenia. They found no difference in the patterns of delusions experienced between those with chronic METH psychosis and schizophrenia and that auditory hallucinations were the most common type of hallucination experienced between groups. However, those with chronic METH psychosis significantly experienced greater visual and tactile hallucinations relative to schizophrenia while those with schizophrenia endorsed greater conceptual disorganization. This suggests that thought disorder may be specific to schizophrenia and not present in either acute or chronic METH psychoses while tactile/visual hallucinations, such as formication, may be more reflective of METH-induced psychoses. These findings are strengthened by the fact that those in the METH psychosis group could only be included if their psychosis occurred after the use of METH, and those in the schizophrenia group could not have a history of drug use disorder, meaning that the diagnosis of each psychiatric condition was independent to the effect of several confounds.

The profiles of acute METH psychosis, persistent METH psychosis and schizophrenia have also been compared. Chen et al. (105) examined the positive symptoms experienced by those with acute METH psychosis (experienced psychosis for <1 month following abstinence), persistent METH psychosis (psychosis presents following abstinence from METH > 1 month) and schizophrenia using the PANNS. Those with persistent METH psychosis and schizophrenia demonstrated comparable severity and frequency of positive symptoms, and both of these groups had PANNS scores that were significantly higher than those in the acute METH psychosis group. These findings may suggest that those with acute METH psychosis may not experience positive symptoms to the same frequency and severity as those with schizophrenia and chronic METH psychosis. However, it should be noted that those in the acute METH group were abstinent for an average of 9 weeks at the time of assessment, and therefore, the results may not truly reflect the severity of these symptoms experienced at the time of their psychotic episodes.

Recently, McKetin et al. (109) classified 284 METH dependent participants as experiencing no current psychotic symptoms, transient psychotic symptoms when using METH, psychotic symptoms during METH use and more than 1 month abstinent (i.e., persistent METH psychosis) or as experiencing primary psychosis (i.e., schizophrenia), and examined the lifetime experience of hallucinations and delusions between groups. Relative to acute METH psychosis, it was shown that persistent METH-induced psychosis was associated with greater lifetime experiences of thought interference and delusions of reference while primary psychosis was likely to experience the same symptoms in addition to thought projection, erotomania, olfactory hallucinations and passivity (relative to acute METH psychosis). Furthermore, those with persistent METH psychosis and schizophrenia also reported reduced symptoms of visual and tactile hallucinations relative to those in the transient METH psychosis group. Importantly, the lifetime delusion and hallucination symptom profiles were not significantly different between persistent METH psychosis and primary psychosis, suggesting that the positive symptoms are comparable between the two conditions. However, it should be noted that those in the primary psychosis group were also METH dependent, suggesting that the results are not independent to drug effects, and as the authors indicate, those in the primary psychosis group may have experienced mania as opposed to schizophrenia. Furthermore, the authors examined the lifetime prevalence of psychotic symptoms, rather than those experienced during their psychotic episodes, which may explain why there was no differences between chronic psychotic syndromes. Nevertheless, these findings suggest that patients who present with greater severity and frequency of lifetime delusions and hallucinations (particularly thought interference, delusions of references and auditory hallucinations) may be at increased risk for the development of recurrent psychotic episodes or a primary psychotic disorder.

## Negative symptoms

An overview of the design and findings of individual research studies that have directly compared negative symptoms in those with METH psychoses to those with schizophrenia can be found in Table 1. The methodological considerations in the examination of research that has compared symptoms between METH psychosis and schizophrenia is shown in Table 2.

### Acute meth psychosis vs. schizophrenia

While stimulant-induced psychotic disorders have been predominantly characterized by positive symptoms, negative symptoms such as flat affect, social withdrawal, apathy, loss of drive, anhedonia and poverty of speech have also been reported in METH psychosis samples (4, 40, 113, 123, 132). Srisurapanont et al. (113) showed no difference between METH psychosis and schizophrenia on measures of psychomotor retardation, flattened affect and poverty of speech using the Manchester scale, while other researchers have found no significant differences between METH psychosis and schizophrenia using the BPRS (107) or the PANSS (111).

However, some researchers have shown differences in the severity of negative symptoms experienced between acute METH psychosis and schizophrenia. For example, Hajebi et al. (117) found that on admission to hospital, those with non-affective psychosis had more severe negative symptoms than those with acute METH-psychosis. Furthermore, while the severity of negative symptoms had improved for both groups upon discharge, the non-affective psychosis group continued to maintain increased severity of negative symptoms relative to the acute METH-psychosis group. There is also indirect evidence that negative symptoms are less severe in acute METH psychosis compared to schizophrenia. Negative symptoms are common in schizophrenia, with negative symptoms considered a central feature of its phenomenology and diagnostic criteria (133, 134). Indeed, 58% of individuals with schizophrenia experience negative symptoms (135), with 50–90% of those with schizophrenia displaying negative symptoms in first-episode psychosis (136). On the other hand (132) found that only 25% of individuals hospitalized with METH psychosis exhibited negative symptoms while (122) similarly found that only 21.4% of their sample met criteria for negative symptoms in a clinical interview using the MINI-plus. While these lower prevalence rates may be attributable to limited research in the area, specifically with respect to inclusion and appropriate assessment of negative symptoms in research studies, these findings suggest that negative symptoms may be experienced at a considerably lower rate in acute METH psychosis compared with schizophrenia.

### Chronic meth psychosis vs. schizophrenia

Previous research has explicitly compared the negative symptoms between chronic METH psychosis and schizophrenia. Tomiyama (114) examined the experience of negative symptoms between 11 participants with chronic METH psychosis and 11 participants with schizophrenia using the Scale for the Assessment of Negative Symptoms (SANS). They found that negative symptoms were milder in chronic METH psychosis overall when compared to schizophrenia. When examining the individual symptoms, however, they found that ratings of avolition-apathy, anhedonia-asociality, and attentional impairment were similar between both groups, but those with schizophrenia demonstrated elevated symptoms of affective flattening and alogia. Additionally, Wang et al. (115) found that schizophrenia was associated with greater frequency and severity of negative symptoms compared to those with chronic METH psychosis. Specifically, those with schizophrenia demonstrated elevated scores for blunted affect, emotional withdrawal and motor retardation. Furthermore, in differentiating between acute and chronic METH psychosis with schizophrenia (105), using the BPRS, found that those with schizophrenia demonstrated the greatest severity of negative symptoms compared to those with acute and chronic METH psychosis, but the negative symptoms demonstrated by the chronic METH psychosis group were significantly greater than those in the acute METH group. Therefore, even though negative symptoms have been reported in both schizophrenia and METH psychosis, schizophrenia appears to be associated with greater prevalence and severity of negative symptoms compared to METH psychoses.

## Cognitive symptoms

An overview of the design and findings of individual research studies that have directly compared the cognitive symptoms associated with METH psychosis to those of schizophrenia can be found in Table 1. The methodological considerations in the examination of research that has compared symptoms between METH psychosis and schizophrenia is shown in Table 2.

### Acute meth psychosis vs. schizophrenia

Recent work has examined the prevalence and severity of cognitive dysfunction following acute METH psychosis in comparison with schizophrenia. Jacobs et al. (108) in an exploratory cross-sectional study, compared the cognitive profile of individuals hospitalized with METH psychosis with patients with paranoid schizophrenia across eight cognitive domains, including premorbid intellectual ability, learning and memory, executive functioning, general intellectual functioning, attention and concentration, motor abilities together with non-verbal and verbal skills. They found no significant differences between the two groups in any cognitive domain examined, suggesting that both METH psychosis and schizophrenia may have similar cognitive profiles and may therefore share underlying brain pathology, particularly with respect to dysfunction of the frontal and temporal lobes. However, there are several limitations to these findings. Firstly, the groups had small sample sizes. Secondly, there were between-group differences in age, ethnicity and place of birth between those with METH psychosis and schizophrenia and as such, these factors may have been confounds in the study. Additionally, it was not known how long the sample had been abstinent from METH nor was it reported how long the METH psychosis sample had been taking METH prior to their participation in the study. Regardless, this initial study suggested that METH psychosis may show cognitive deficits, similar to those typically reported in schizophrenia.

Ezzatpanah et al. (106) further compared cognitive function in individuals with METH-induced psychosis and schizophrenia to healthy controls, with all subjects matched for age, sex and education. They found that both METH psychosis and schizophrenia were characterized by reduced performance on all cognitive tasks examined when compared to healthy controls, and there were no significant differences in the performance of those with acute METH psychosis and schizophrenia across tasks of memory, sustained attention, selective attention and executive functioning. Specifically, METH psychosis and schizophrenia groups demonstrated difficulty in inhibiting, manipulating and suppressing information, together with difficulties learning and retaining verbal information over time. These findings indicate that both disorders may be characterized by comparable deficits of cognition mediated by the temporal and frontal lobes, specifically the prefrontal cortex, and further extends the findings of Jacobs et al. (108) that both METH psychosis and schizophrenia may be the product of similar pattern of brain pathology. While both these studies were hampered by small sample sizes, these similar findings are strengthened by the fact that these two studies were derived from different cultural samples - American (108) and Iranian (106)–and through the use of distinct cognitive measures. However, despite the overwhelming similarities in cognitive dysfunction between METH psychosis and schizophrenia (106) found that individuals with schizophrenia and METH psychosis demonstrated difficulties with sustained visual attention compared to controls, yet those with schizophrenia performed worse than subjects with acute METH psychosis. As selective visual attention is primarily correlated with the parietal cortex (137), these findings indicate that dysfunction of the parietal cortex may be more pronounced in schizophrenia than acute METH psychosis.

### Chronic meth psychosis vs. schizophrenia

The above studies were based on recent abstinent METH users and may not be generalizable to those with chronic METH psychosis. However, there is emerging evidence of similarities between cognitive symptoms in persistent METH psychosis and schizophrenia in the literature. For example, (116) examined individuals with METH psychosis and schizophrenia on a verbal fluency tasks while they had their brain blood oxygenation levels recorded using Functional Near-Infrared Spectroscopy (NIRS). They also had their cognitive ability measured using the Brief Assessment of Cognition in Schizophrenia (BASC). They found there was no difference between those with METH-psychosis and schizophrenia on tasks of verbal memory, working memory, motor speed, verbal fluency, attention, speed of information processing, executive functioning and total cognitive ability. However, oxyhaemaoglobin changes in the prefrontal cortex were higher in the METH psychosis group compared to schizophrenia, particularly in the right dorsolateral prefrontal cortex. This suggests that while the cognitive ability may be similarly perturbed across METH psychosis and schizophrenia, there are biological changes that may be used to distinguish between the two conditions. However, a significant limitation in this study is that the length of abstinence for the METH psychosis group was not stated. Given that the sample had been 8.42 years since the onset of psychotic symptoms (average of 2.5 hospitalizations and 8.42 months of hospitalization), the sample likely reflects a more persistent METH psychosis, and the researchers referred to the sample as “methamphetamine-induced psychotic disorder.” However, in the absence of abstinence information, it is uncertain whether these findings are applicable to acute or chronic METH psychosis, or the sample could reflect a blended representation.

More recent research has shown that cognitive dysfunction is specific to the persistent form of METH psychosis rather than acute METH psychosis. For example, Chen et al. (105) conducted a well-designed cross-sectional study on healthy controls, METH users without psychosis, METH users with acute psychosis (METH users who had psychotic symptoms that dissipated within 1 month following abstinence), METH users with persistent psychosis (psychosis greater than 1 month), and individuals with schizophrenia. They found that METH users with persistent psychosis performed comparably to those with schizophrenia across all cognitive domains, with both these groups performing worse than the other acute METH psychosis, METH users without psychosis and control groups. These findings extend the findings of Jacobs et al. (108) and Ezzatpanah et al. (106) by clearly distinguishing between METH users with acute and persistent psychoses, suggesting that schizophrenia and only persistent psychosis secondary to METH use are associated with similar cognitive profiles. This may indicate that the samples used in previous studies could represent a mixture of both acute and persistent METH psychoses. These findings therefore suggest that cognitive dysfunction may develop in individuals who originally had acute symptoms that endured over time, likely as a secondary consequence to neuropathological changes that coincide with abstinence. Altogether, these findings indicate that chronic METH-induced psychosis is associated with brain changes that may be carefully distinguished from the changes concomitant with METH use and acute METH psychosis.

## Methodological limitations and considerations

An overview of the methodological considerations in the examination of research that has compared symptoms between METH psychosis and schizophrenia is shown in Table 2. One of the biggest limitations with METH psychosis research is that little effort is made to distinguish between those with acute and chronic METH psychosis, with the majority of the findings portraying a blended representation of all types. Indeed, the majority of studies failed to report the length of time of abstinence from METH at the time of the assessment. For the purpose of this review, therefore, we considered these studies to represent acute METH psychosis studies as there was no evidence to suggest that these samples had been abstinent for long enough to be considered to be representative of chronic METH psychosis [>1 month abstinence according to published papers, e.g., (105)]. Additionally, few studies indicate whether their samples are abstinent at the time of the assessment. Taken together, the findings presented in these papers may not be generalizable to samples of chronic METH psychosis, as it is uncertain whether these behavioral outcomes are referable to the direct effects of METH, acute METH psychosis or persistent METH psychosis.

In keeping with this limitation, some studies implement diagnostic criteria for chronic METH psychosis that is distinct by those provided by the DSM-5. That is, these studies typically categorize those who continue to experience psychosis after discontinuing METH for more than 1 month as those with persistent METH psychosis. According to the DSM, however, these patients should be diagnosed with a primary psychotic disorder, and some studies adhere to these guidelines. Consequently, individuals with chronic METH psychosis may be categorized as participants with schizophrenia. There are several implications to this procedure. Firstly, chronic METH psychosis may be underreported across scientific literature. Secondly, the inappropriate allocation of participants to treatment conditions precludes the examination of distinct clinical and symptom profiles between conditions given the significant heterogeneity across various outcome measures. Furthermore, inconsistency in sample characteristics hinders the ability to pool data and conclusions across research studies. Indeed, it is worth noting that other researchers have proposed similar concerns about this approach to METH psychosis research (138). Given that only METH users with a persistent METH psychosis syndrome appear to display the cognitive dysfunction typically associated with schizophrenia (105), it is possible that the differences in positive, negative and cognitive symptoms reported in additional studies may refer only to acute METH psychosis. It will be important for future research to examine the effect of persistent METH psychosis and how this relates to the behavioral, cognitive and biological changes typically reported in schizophrenia in order to elucidate whether chronic METH psychosis represents a distinct psychotic disorder and to differentiate the clinical profiles of acute vs. chronic psychosis forms.

There are additional limitations to this field of research that should be addressed. Firstly, many studies are of low sample size, meaning that many of the similarities between METH psychosis and schizophrenia may be due to low statistical power to detect significant difference between groups. Secondly, many studies do not control for polydrug use, meaning that the symptoms of psychosis may not be exclusively attributed to METH administration. Thirdly, studies do not actively control for the effect of psychotropic medication, particularly given that this impacts on the presentation of behavioral symptoms. Additionally, many studies are reliant on hospitalized samples, which are likely concomitant with more severe psychosis than non-treatment-seeking individuals with METH psychosis in the community. Lastly, many studies compare METH psychosis to schizophrenia using screening or brief assessment tools. A significant limitation of these scales is that they do not differentiate the qualitative nature of the hallucinations or delusions experienced, as they quantify the status of positive symptoms with a total score, meaning that these scales may be unable to detect differences that may differentiate these conditions. The use of such tools may explain why research studies produce such contrasting, and at times conflicting, clinical profiles. For example, the positive symptoms associated with METH psychosis and schizophrenia using the PANSS and BPRS are comparable, but examination using more indepth tools, such as the Manchester Scale, revealed differences in the type of positive symptoms experienced between groups.

## Summary and conclusions

**Figure 1:**
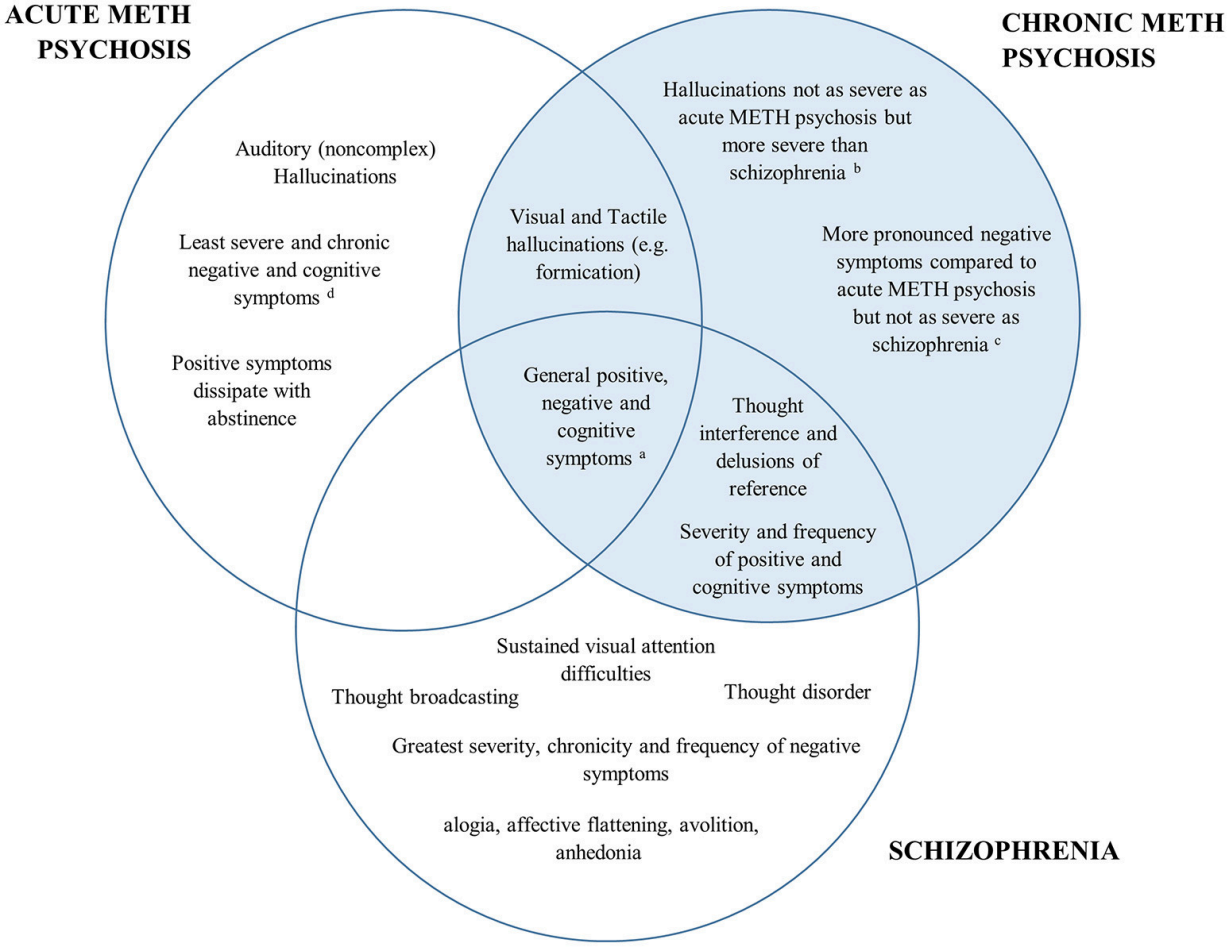
Venn diagram of the overlap in psychiatric and cognitive symptomatology between acute METH psychosis, chronic METH psychosis, and schizophrenia. The left represents symptoms specific to acute METH psychosis, the right (highlighted blue) represents the symptoms and profile specific to chronic METH psychosis, while the bottom highlights those associated specifically with a schizophrenia profile. Symptoms that are common across disorders are shown in the overlap. ^a^All conditions demonstrate some degree of positive, negative and cognitive symptomatology according the specific syndrome scales (e.g., Brief Psychiatric Rating Scale or the Positive and Negative Severity Scale). ^b^Visual and tactile hallucinations: Acute METH psychosis > Chronic METH psychosis > Schizophrenia ^c^Severity of negative symptoms: Schizophrenia > Chronic METH psychosis > Acute METH psychosis. ^d^Cognition: Schizophrenia = Chronic METH psychosis > Acute METH psychosis.

A comparison of the positive, negative and cognitive symptoms between schizophrenia and acute/chronic METH psychosis is detailed in Figure 1. Research has shown both similarities and differences in the positive, negative and cognitive symptoms between METH-induced psychosis and schizophrenia. There appears to be a high degree of concordance in the type, prevalence and severity of positive symptoms between METH-induced psychosis and schizophrenia, confirming that it would be difficult to distinguish between the two conditions in the clinical setting based on the positive symptoms alone. However, while auditory hallucinations appear to be the most common hallucination reported in METH psychosis (acute and chronic) and schizophrenia, visual and tactile hallucinations appear to be more prominent in acute/transient METH psychosis, with thought disorder the most pronounced symptom in schizophrenia. While negative symptoms occur in both conditions, some research has indicated that there are differences in the type, severity and progression of negative symptoms throughout both conditions, with METH psychosis associated with reduced frequency and severity of several negative markers, such as flattened affect (although chronic psychosis is associated with worse negative symptoms than acute METH psychosis). Lastly, from a cognitive perspective, most cognitive domains appear to be similarly perturbed across METH psychosis and schizophrenia. However, recent findings have highlighted that some functions subserved by the parietal cortex, such as selective visual attention, may be more pronounced in schizophrenia that acute METH psychosis, and that cognitive dysfunction may be specifically comparable to schizophrenia for those with chronic METH psychosis.

Overall, while there is considerable overlap in the behavioral and cognitive symptoms between acute METH psychosis and schizophrenia, research has shown that there are unique aspects to each condition. While both disorders may be characterized by common underlying biological pathologies and phenotypes, acute METH psychosis could represent a distinct psychotic disorder to schizophrenia and may be clinically distinguished from a primary psychotic disorder based on distinct behavioral and cognitive sequelae. On the other hand, preliminary evidence suggests that chronic METH psychosis may be clinically similar to that of primary psychotic disorders, particularly with respect to positive and cognitive symptomatology. However, given the number of limitations evident in the available studies, particularly with respective to the paucity of experimental designs that differentiate between acute and chronic forms of METH psychosis, there is insufficient evidence to conclude whether chronic METH psychosis is clinically distinct from schizophrenia.

Nevertheless, these findings may have implications for the longer-term management and treatment of such conditions. For example, concerning the management of acute METH psychosis, symptoms will resolve with abstinence from METH and with the appropriate management of withdrawal. Therefore, for the most part, long-term pharmacological interventions for acute METH psychoses would not be needed or beneficial (139). However, given the similarity in symptoms between persistent METH psychosis and schizophrenia, second generation antipsychotic medicines, such as risperidone and olanzapine, may be appropriate intervention strategies. While first-generation anti-psychotics (i.e., haloperidol) may useful for the management of schizophrenia, and therefore, persistent METH psychosis, such medicines are at elevated risk of causing extrapyramidal symptoms in individuals with METH induced psychosis, and should therefore be used carefully (56, 140). However, these suggestions are not clinical recommendations and should be further examined using large randomized clinical trials so that clinical guidelines on the appropriate treatment of these conditions can be developed.

## Author contributions

TW and JC designed the review. TW conducted the review; TW wrote the initial version of the manuscript with subsequent contribution from JC.

### Conflict of interest statement

The authors declare that the research was conducted in the absence of any commercial or financial relationships that could be construed as a potential conflict of interest. The handling Editor declared a shared affiliation, though no other collaboration, with one of the authors TW at the time of the review.

## References

[1] Anglin MD Burke C Perrochet B Stamper E Dawud-Noursi S. History of the methamphetamine problem. J Psychoactive Drugs (2000) 32:137–41. 10.1080/02791072.2000.10400221 10908000

[2] Hart CL Marvin CB Silver R Smith EE. Is cognitive functioning impaired in methamphetamine users? A critical review. Neuropsychopharmacology (2012) 37:586–608. 10.1038/npp.2011.276 22089317 PMC3260986

[3] Meredith CW Jaffe C Ang-Lee K Saxon AJ. Implications of chronic methamphetamine use: a literature review. Harv Rev Psychiatry (2005) 13:141–54. 10.1080/10673220591003605 16020027

[4] Zorick TS Rad D Rim C Tsuang J An overview of methamphetamine-induced psychotic syndromes. Addict Disord Their Treatm. (2008) 7:143–56. 10.1097/ADT.0b013e318066d5e0

[5] Homer BD Solomon TM Moeller RW Mascia A DeRaleau L Halkitis PN. Methamphetamine abuse and impairment of social functioning: a review of the underlying neurophysiological causes and behavioral implications. Psychol Bull. (2008) 134:301–10. 10.1037/0033-2909.134.2.301 18298273

[6] Chuang L-W Karoum F Jed Wyatt R. Different effects of behaviorally equipotent doses of amphetamine and methamphetamine on brain biogenic amines: specific increase of phenylethylamine by amphetamine. Euro J Pharmacol. (1982) 81:385–92. 10.1016/0014-2999(82)90103-0 7117382

[7] Fleckenstein AE Volz TJ Riddle EL Gibb JW Hanson GR. New insights into the mechanism of action of amphetamines. Annu Rev Pharmacol Toxicol. (2007) 47:681–98. 10.1146/annurev.pharmtox.47.120505.105140 17209801

[8] Freye E Pharmacology of methamphetamine. In: Fry E Levi J, Editors. Pharmacology and Abuse of Cocaine, Amphetamines, Ecstasy and Related Designer Drugs. Dordrecht: Springer (2010). pp. 119–24. 10.1007/978-90-481-2448-0

[9] Izawa J Yamanashi K Asakura T Misu Y Goshima Y. Differential effects of methamphetamine and cocaine on behavior and extracellular levels of dopamine and 3,4-dihydroxyphenylalanine in the nucleus accumbens of conscious rats. Eur J Pharmacol. (2006) 549:84–90. 10.1016/j.ejphar.2006.08.031 16979160

[10] Weisheit R Methamphetamine: Its History, Pharmacology and Treatment. Minnesota: Hazelden Publishing (2013).

[11] Elliott JM Beveridge TJ. Psychostimulants and monoamine transporters: upsetting the balance. Curr Opin Pharmacol. (2005) 5:94–100. 10.1016/j.coph.2004.09.005 15661632

[12] Pierce RC Kalivas PW. A circuitry model of the expression of behavioral sensitization to amphetamine-like psychostimulants. Brain Res Brain Res Rev. (1997) 25:192–216. 9403138 10.1016/s0165-0173(97)00021-0

[13] UNODC World Drug Report. New York, NY: UNODC (2009).

[14] UNODC Global Amphetamine-type Stimulant Assessment: Amphetamine and Ecstasy. New York, NY: UNODC (2011).

[15] McKetin R Lubman DI Baker AL Dawe S Ali RL. Dose-related psychotic symptoms in chronic methamphetamine users: evidence from a prospective longitudinal study. JAMA Psychiatry (2013) 70:319–24. 10.1001/jamapsychiatry.2013.283 23303471

[16] UNODC World Drug Report. New York, NY: UNODC (2013).

[17] UNODC World Drug Report. from New York, NY: UNODC (2014).

[18] UNODC World Drug Report. New York, NY: UNODC (2003).

[19] UNODC Global Synthetic Drugs Assessment: Amphetamine-Type Stimulants and New Psychoactive Substances. New York, NY: UNODC (2014).

[20] SAMHSA National Admissions to Substance Abuse Treatment Service, Treatment Episode Data Set (TEDS): 1994-2004. Rockville, MD: Office of Applied Studies (2006).

[21] SAMHSA Reults from the 2007 National Survey on Drug Use and Health: National Findings. Rockville, MD: Office of Applied Studies (2008).

[22] SAMHSA Treatment Episode Data Set (TEDS): 2004–2014. National Admissions to Substance Abuse Treatment Services. No. BHSIS Series S-84, HHS Publication No. SMA (2016). pp. 16–4986.

[23] AIHW Alcohol and Other Drug Treatment Services in Australia 2014-15. Canberra, ACT: AIHW (2016).

[24] AIHW National Hospital Morbidity Database: Principal Diagnosis Data Cubes for 1993-94 to 2012-13. Canberra, ACT: AIHW (2016).

[25] QLDHealth Queensland Methamphetamine Paper. Brisbane, QLD: QLDHealth (2017).

[26] Cook CE Jeffcoat AR Hill JM Pugh DE Patetta PK Sadler BM. Pharmacokinetics of methamphetamine self-administered to human subjects by smoking S-(+)-methamphetamine hydrochloride. Drug Metab Disposition (1993) 21:717–23. 8104133

[27] Cruickshank CC Dyer KR. A review of the clinical pharmacology of methamphetamine. Addiction (2009) 104:1085–99. 10.1111/j.1360-0443.2009.02564.x 19426289

[28] Topp L Degenhardt L Kaye S Darke S. The emergence of potent forms of methamphetamine in Sydney, Australia: a case study of the IDRS as a strategic early warning system. Drug Alcohol Rev. (2002) 21:341–8. 10.1080/0959523021000023199 12537703

[29] AIHW National Drug Strategy Household Survey Report. Canberra, ACT: AIHW (2010).

[30] AIHW National Drug Strategy Household Survey Report. Canberra: ACT: AIHW (2013).

[31] Bramness J Gundersen O Guterstam J Rognli E Konstenius M Loberg E-M. Amphetamine-induced psychosis - a separate diagnostic entity or primary psychosis triggered in the vulnerable? BMC Psychiatry (2012) 12:221. 10.1186/1471-244X-12-221 23216941 PMC3554477

[32] McKetin R McLaren J Lubman DI Hides L. The prevalence of psychotic symptoms among methamphetamine users. Addiction (2006) 101:1473–8. 10.1111/j.1360-0443.2006.01496.x 16968349

[33] Zweben JE Cohen JB Christian D Galloway GP Salinardi M Parent D. Psychiatric symptoms in methamphetamine users. Am J Addict. (2004) 13:181–90. 10.1080/10550490490436055 15204668

[34] Sato M. A lasting vulnerability to psychosis in patients with previous methamphetamine psychosis. Ann N Y Acad Sci. (1992) 654:160–70. 1632581 10.1111/j.1749-6632.1992.tb25965.x

[35] Ujike H Sato M. Clinical features of sensitization to methamphetamine observed in patients with methamphetamine dependence and psychosis. Ann N Y Acad Sci. (2004) 1025:279–87. 10.1196/annals.1316.035 15542728

[36] Salo R Fassbender C Iosif AM Ursu S Leamon MH Carter C. Predictors of methamphetamine psychosis: history of ADHD-relevant childhood behaviors and drug exposure. Psychiatry Res. (2013) 210:529–35. 10.1016/j.psychres.2013.06.030 23896355 PMC3818411

[37] Lecomte T Dumais A Dugré JR Potvin S. The prevalence of substance-induced psychotic disorder in methamphetamine misusers: a meta-analysis. Psychiatry Res. (2018) 268:189–92. 10.1016/j.psychres.2018.05.033 30041133

[38] Mahoney J Hawkins R De La Garza R Kalechstein A Newton T. Relationship between gender and psychotic symptoms in cocaine-dependent and methamphetamine-dependent participants. Gend Med. (2010) 7:414–21. 10.1016/j.genm.2010.09.003 21056868 PMC3342664

[39] McKetin R Hickey K Devlin K Lawrence K. The risk of psychotic symptoms associated with recreational methamphetamine use. Drug Alcohol Rev. (2010) 29:358–63. 10.1111/j.1465-3362.2009.00160.x 20636650

[40] Chen CK Lin SK Sham PC Ball D Loh EW Hsiao CC. Pre-morbid characteristics and co-morbidity of methamphetamine users with and without psychosis. Psychol Med. (2003) 33:1407–14. 10.1017/S0033291703008353 14672249

[41] McKetin R Kelly E McLaren J. The relationship between crystalline methamphetamine use and methamphetamine dependence. Drug Alcohol Depend. (2006) 85:198–204. 10.1016/j.drugalcdep.2006.04.007 16723192

[42] Matsumoto T Kamijo A Miyakawa T Endo K Yabana T Kishimoto H. Methamphetamine in Japan: the consequences of methamphetamine abuse as a function of route of administration. Addiction (2002) 97:809–817. 10.1046/j.1360-0443.2002.00143.x 12133119

[43] Lappin JM Roxburgh A Kaye S Chalmers J Sara G Dobbins T. Increased prevalence of self-reported psychotic illness predicted by crystal methamphetamine use: evidence from a high-risk population. Int J Drug Policy (2016) 38:16–20. 10.1016/j.drugpo.2016.10.018 27842249

[44] Farrell M Boys A Bebbington P Brugha T Coid J Jenkins R. Psychosis and drug dependence: results from a national survey of prisoners. Br J Psychiatry (2002) 181:393–8. 10.1192/bjp.181.5.393 12411264

[45] McKetin R Estimating the Number of Regular and Dependent Methamphetamine Users in Australia. Sydney: National Drug and Alcohol Research Centre (2005).

[46] Degenhardt L Sara G McKetin R Roxburgh A Dobbins T Farrell M. Crystalline methamphetamine use and methamphetamine-related harms in *Australia*. Drug Alcohol Rev. (2017) 36:160–70. 10.1111/dar.12426 27286742

[47] Richards JR Hamidi S Grant CD Wang CG Tabish N Turnipseed SD. Methamphetamine use and emergency department utilization: 20 years later. J Addict. (2017) 2017:4050932. 10.1155/2017/4050932 28913001 PMC5585625

[48] Alam Mehrjerdi Z Noroozi A. Methamphetamine Intoxication in Emergency Departments of Hospitals in Iran: implications for treatment. Iran J Med Sci. (2013) 38:347–8. 24293791 PMC3838989

[49] Deng X Huang Z Li X Li Y Wang Y Wu D. Long-term follow-up of patients treated for psychotic symptoms that persist after stopping illicit drug use. Shang Archiv Psychiatry (2012) 24:271–8. 10.3969/j.issn.1002-0829.2012.05.004 25328350 PMC4198875

[50] Iwanami A Sugiyama A Kuroki N Toda S Kato N Nakatani Y. Patients with methamphetamine psychosis admitted to a psychiatric hospital in Japan. A preliminary report. Acta Psychiatr Scand. (1994) 89:428–32. 8085475 10.1111/j.1600-0447.1994.tb01541.x

[51] Nakatani Y Yoshizawa F Yamada H Iwanami A Sakaguchi M Katoh N. Methamphetamine psychosis in Japan: a survey. Br J Addict. (1989) 84:1548–9. 2611442 10.1111/j.1360-0443.1989.tb03941.x

[52] Teraoka A. [Prognostic and criminological studies on the mental disorder caused by the chronic methamphetamine intoxication]. Seishin Shinkeigaku Zasshi (1967) 69:597–619. 5625393

[53] Glasner-Edwards S Mooney LJ Marinelli-Casey P Hillhouse M Ang A Rawson RA. Psychopathology in methamphetamine-dependent adults 3 years after treatment. Drug Alcohol Rev (2010) 29:12–20. 10.1111/j.1465-3362.2009.00081.x 20078677 PMC3772133

[54] Grelotti D Kanayama G Pope H. Remission of persistent methamphetamine-induced psychosis after electroconvulsive therapy: presentation of a case and review of the literature. Am J Psychiatry (2010) 167:17–23. 10.1176/appi.ajp.2009.08111695 20068123

[55] Arunogiri S Foulds JA McKetin R Lubman DI. A systematic review of risk factors for methamphetamine-associated psychosis. Aust N Z J Psychiatry (2018) 52:514–29. 10.1177/0004867417748750 29338289

[56] Cadet JL Gold MS Methamphetamine-induced psychosis: who says all drug use is reversible? Curr. Psychiatry (2018) 16:15–20.

[57] Glasner-Edwards S Mooney LJ. Methamphetamine psychosis: epidemiology and management. CNS Drugs (2014) 28:1115–26. 10.1007/s40263-014-0209-8 25373627 PMC5027896

[58] Grant KM LeVan TD Wells SM Li M Stoltenberg SF Gendelman HE. Methamphetamine-associated psychosis. J Neuroimmune Pharmacol. (2012) 7:113–139. 10.1007/s11481-011-9288-1 21728034 PMC3280383

[59] McKetin R. Methamphetamine psychosis: insights from the past. Addiction (2018) 113:1522–7. 10.1111/add.14170 29516555

[60] Suetani S Reddan J Anderson C. Methamphetamine and psychiatry: a story of the colourless substance of abuse. Aus Psychiatry (2017) 25:254–6. 10.1177/1039856217695702 28541728

[61] Keefe R. Should cognitive impairment be included in the diagnostic criteria for schizophrenia? World Psychiatry (2008) 7:22–8. 10.1002/j.2051-5545.2008.tb00142.x 18458774 PMC2327232

[62] Blanchard JJ Cohen AS. The structure of negative symptoms within schizophrenia: implications for assessment. Schizophrenia Bull. (2006) 32:238–45. 10.1093/schbul/sbj013 16254064 PMC2632211

[63] Liemburg E Castelein S Stewart R van der Gaag M Aleman A Knegtering H. Two subdomains of negative symptoms in psychotic disorders: established and confirmed in two large cohorts. J Psychiatric Res. (2013) 47:718–25. 10.1016/j.jpsychires.2013.01.024 23472837

[64] Breier A Schreiber JL Dyer J Pickar D. National Institute of Mental Health longitudinal study of chronic schizophrenia. Prognosis and predictors of outcome. Arch Gen Psychiatry (1991) 48:239–46. 1671741 10.1001/archpsyc.1991.01810270051007

[65] Association AP Diagnostic and Statistical Manual of Mental Disorders, (DSM-5®). Arlington: American Psychiatric Publication (2013).

[66] Aleman A Hijman R de Haan EH Kahn RS. Memory impairment in schizophrenia: a meta-analysis. Am J Psychiatry (1999) 156:1358–66. 10484945 10.1176/ajp.156.9.1358

[67] Elvevag B Goldberg TE. Cognitive impairment in schizophrenia is the core of the disorder. Crit Rev Neurobiol. (2000) 14:1–21. 10.1615/CritRevNeurobiol.v14.i1.10 11253953

[68] Goldberg TE Gold JM Neurocognitive functioning in patients with schizophrenia: an overview. In: Bloom FE Kupfer DJ Editors. Psychopharmacology: The Fourth Generation of Progress. New York, NY: Raven Press (1995). pp. 245–1257.

[69] Green MF Kern RS Heaton RK. Longitudinal studies of cognition and functional outcome in schizophrenia: implications for MATRICS. Schizophrenia Res. (2004) 72:41–51. 10.1016/j.schres.2004.09.009 15531406

[70] Heinrichs RW Zakzanis KK. Neurocognitive deficit in schizophrenia: a quantitative review of the evidence. Neuropsychology (1998) 12:426–45. 9673998 10.1037//0894-4105.12.3.426

[71] Weickert TW Goldberg TE Gold JM Bigelow LB Egan MF Weinberger DR. Cognitive impairments in patients with schizophrenia displaying preserved and compromised intellect. Archiv Gen Psychiatry (2000) 57:907–13. 10.1001/archpsyc.57.9.907 10986554

[72] Bozikas VP Andreou C. Longitudinal studies of cognition in first episode psychosis: a systematic review of the literature. Aust N Z J Psychiatry (2011) 45:93–108. 10.3109/00048674.2010.541418 21320033

[73] Dawes SE Jeste DV Palmer BW. Cognitive profiles in persons with chronic schizophrenia. J Clin Exper Neuropsychol. (2011) 33:929–36. 10.1080/13803395.2011.578569 21644139 PMC3221409

[74] Lewandowski KE Cohen BM Öngur D. Evolution of neuropsychological dysfunction during the course of schizophrenia and bipolar disorder. Psychol Med. (2011) 41:225–41. 10.1017/S0033291710001042 20836900

[75] Szöke A Trandafir A Dupont M-E Méary A Schürhoff F Leboyer M. Longitudinal studies of cognition in schizophrenia: meta-analysis. Br J Psychiatry (2008) 192:248–57. 10.1192/bjp.bp.106.029009 18378982

[76] Barder HE Sundet K Rund BR Evensen J Haahr U Ten Velden Hegelstad W. Ten year neurocognitive trajectories in first-episode psychosis. Front Hum Neurosci. (2013) 7:643. 10.3389/fnhum.2013.00643 24109449 PMC3791439

[77] Mesholam-Gately RI Giuliano AJ Goff KP Faraone SV Seidman LJ. Neurocognition in first-episode schizophrenia: a meta-analytic review. Neuropsychology (2009) 23:315–36. 10.1037/a0014708 19413446

[78] Saykin AJ Shtasel DL Gur RE Kester DB Mozley LH Stafiniak P. Neuropsychological deficits in neuroleptic naive patients with first-episode schizophrenia. Arch Gen Psychiatry (1994) 51:124–31. 7905258 10.1001/archpsyc.1994.03950020048005

[79] Øie M Sundet K Rund BR. Neurocognitive decline in early-onset Schizophrenia compared with ADHD and normal controls: evidence from a 13-year follow-up study. Schizophr Bull. (2010) 36:557–65. 10.1093/schbul/sbn127 18801881 PMC2879697

[80] Goff DC Hill M Barch D. The treatment of cognitive impairment in schizophrenia. Pharmacol Biochem Behav. (2011) 99:245–53. 10.1016/j.pbb.2010.11.009 21115035 PMC3114283

[81] Hutton S Puri B Duncan L-J Robbins T Barnes T Joyce E. Executive function in first-episode schizophrenia. Psychol Med. (1998) 28:463–73. 9572103 10.1017/s0033291797006041

[82] Minzenberg MJ Laird AR Thelen S Carter CS Glahn DC. Meta-analysis of 41 functional neuroimaging studies of executive function in schizophrenia. Arch Gen Psychiatry (2009) 66:811–22. 10.1001/archgenpsychiatry.2009.91 19652121 PMC2888482

[83] Mosiołek A Gierus J Koweszko T Szulc A. Cognitive impairment in schizophrenia across age groups: a case–control study. BMC Psychiatry (2016) 16:37. 10.1186/s12888-016-0749-1 26908293 PMC4765123

[84] Rodriguez-Jimenez R Bagney A Martinez-Gras I Ponce G Sanchez-Morla EM Aragues M. Executive function in schizophrenia: influence of substance use disorder history. Schizophr Res. (2010) 118:34–40. 10.1016/j.schres.2009.09.025 19854622

[85] Milev P Ho B-C Arndt S Andreasen NC. Predictive values of neurocognition and negative symptoms on functional outcome in schizophrenia: a longitudinal first-episode study with 7-year follow-up. Am J Psychiatry (2005) 162:495–506. 10.1176/appi.ajp.162.3.495 15741466

[86] Sharma T Antonova L. Cognitive function in schizophrenia. Deficits, functional consequences, and future treatment. Psychiatr Clin North Am. (2003) 26:25–40. 10.1016/S0193-953X(02)00084-9 12683258

[87] Dodge HH Du Y Saxton JA Ganguli M. Cognitive domains and trajectories of functional independence in nondemented elderly persons. J Gerontol A Biol Sci Med Sci. (2006) 61:1330–7. 10.1093/gerona/61.12.1330 17234830 PMC1808540

[88] MacNeill SE Lichtenberg PA. Home alone: the role of cognition in return to independent living. Archiv Phys Med Rehabilit. (1997) 78:755–8. 10.1016/S0003-9993(97)90085-X 9228880

[89] Jaeger J Berns S Loftus S Gonzalez C Czobor P. Neurocognitive test performance predicts functional recovery from acute exacerbation leading to hospitalization in bipolar disorder. Bipolar Disord. (2007) 9:93–102. 10.1111/j.1399-5618.2007.00427.x 17391353

[90] Fillit HM Butler RN O'Connell AW Albert MS Birren JE Cotman CW. Achieving and maintaining cognitive vitality with aging. Mayo Clin Proc. (2002) 77:681–96. 10.4065/77.7.681 12108606

[91] Kittirattanapaiboon P Mahatnirunkul S Booncharoen H Thummawomg P Dumrongchai U Chutha W. Long-term outcomes in methamphetamine psychosis patients after first hospitalisation. Drug Alcohol Rev. (2010) 29:456–61. 10.1111/j.1465-3362.2010.00196.x 20636664

[92] Alderson HL Semple DM Blayney C Queirazza F Chekuri V Lawrie SM. Risk of transition to schizophrenia following first admission with substance-induced psychotic disorder: a population-based longitudinal cohort study. Psychol Med. (2017) 47:2548–55. 10.1017/s0033291717001118 28464965

[93] Niemi-Pynttari JA Sund R Putkonen H Vorma H Wahlbeck K Pirkola SP. Substance-induced psychoses converting into schizophrenia: a register-based study of 18,478 Finnish inpatient cases. J Clin Psychiatry (2013) 74:e94–99. 10.4088/JCP.12m07822 23419236

[94] Callaghan R Cunningham J Allebeck P Arenovich T Sajeev G Remington G. Methamphetamine use and schizophrenia: a population-based cohort study in California. Am J Psychiatry (2012) 169:389–96. 10.1176/appi.ajp.2011.10070937 22193527

[95] Connell PH Amphetamine Psychosis. London: Chapman & Hall (1958).

[96] Bousman CA Glatt SJ Everall IP Tsuang MT. Genetic association studies of methamphetamine use disorders: A systematic review and synthesis. Am J Med Genet B Neuropsychiatr Genet. (2009) 150B:1025–49. 10.1002/ajmg.b.30936 19219857

[97] Ikeda M Okahisa Y Aleksic B Won M Kondo N Naruse N. Evidence for shared genetic risk between methamphetamine-induced psychosis and schizophrenia. Neuropsychopharmacology (2013) 38:1864–70. 10.1038/npp.2013.94 23594818 PMC3746703

[98] Chen C Lin S Sham P Ball D Loh E Murray R. Morbid risk for psychiatric disorder among the relatives of methamphetamine users with and without psychosis. Am J Med Genet B Neuropsychiatr Genet. (2005) 136B:87–91. 10.1002/ajmg.b.30187 15892150

[99] Flaum M Schultz SK. When does amphetamine-induced psychosis become schizophrenia? Am J Psychiatry (1996) 153:812–5. 8633695 10.1176/ajp.153.6.812

[100] Yeh H Lee Y Sun H Wan S. Six months follow-up of patients with methamphetamine psychosis. Zhonghua Yi Xue Za Zhi (2001) 64:388–394. 11584576

[101] Yui K Goto K Ikemoto S Ishiguro T. Stress induced spontaneous recurrence of methamphetamine psychosis: the relation between stressful experiences and sensitivity to stress. Drug Alcohol Depend (2000) 58:67–75. 10.1016/S0376-8716(99)00060-5 10669056

[102] Nordahl TE Salo R Leamon M. Neuropsychological effects of chronic methamphetamine use on neurotransmitters and cognition: a review. J Neuropsychiatry Clin Neurosci. (2003) 15:317–25. 10.1176/jnp.15.3.317 12928507

[103] Potvin S Pelletier J Grot S Hebert C Barr AM Lecomte T. Cognitive deficits in individuals with methamphetamine use disorder: a meta-analysis. Addict Behav. (2018) 80:154–60. 10.1016/j.addbeh.2018.01.021 29407687

[104] Scott JC Woods SP Matt GE Meyer RA Heaton RK Atkinson JH. Neurocognitive effects of methamphetamine: a critical review and meta-analysis. Neuropsychol Rev. (2007) 17:275–97. 10.1007/s11065-007-9031-0 17694436

[105] Chen CK Lin SK Chen YC Huang MC Chen TT Ree SC. Persistence of psychotic symptoms as an indicator of cognitive impairment in methamphetamine users. Drug Alcohol Depend (2015) 148:158–64. 10.1016/j.drugalcdep.2014.12.035 25601645

[106] Ezzatpanah Z Shariat SV Tehrani-Doost M. Cognitive functions in methamphetamine induced psychosis compared to schizophrenia and normal subjects. Iran J Psychiatry (2014) 9:152–7. 25561956 PMC4277805

[107] Hides L Dawe S McKetin R Kavanagh DJ Young RM Teesson M. Primary and substance-induced psychotic disorders in methamphetamine users. Psychiatry Res. (2015) 226:91–6. 10.1016/j.psychres.2014.11.077 25677394

[108] Jacobs E Fujii D Schiffman J Bello I. An exploratory analysis of neurocognition in methamphetamine-induced psychotic disorder and paranoid schizophrenia. Cogn Behav Neurol (2008) 21:98–103. 10.1097/WNN.0b013e31816bdf90 18541986

[109] McKetin R Baker AL Dawe S Voce A Lubman DI. Differences in the symptom profile of methamphetamine-related psychosis and primary psychotic disorders. Psychiatry Res. (2017) 251:349–54. 10.1016/j.psychres.2017.02.028 28282630

[110] Medhus S Mordal J Holm B Morland J Bramness JG. A comparison of symptoms and drug use between patients with methamphetamine associated psychoses and patients diagnosed with schizophrenia in two acute psychiatric wards. Psychiatry Res. (2013) 206:17–21. 10.1016/j.psychres.2012.09.023 23036490

[111] Okada N Takahashi K Nishimura Y Koike S Ishii-Takahashi A Sakakibara E. Characterizing prefrontal cortical activity during inhibition task in methamphetamine-associated psychosis versus schizophrenia: a multi-channel near-infrared spectroscopy study. Addic. Biol. (2016) 21:489–503. 10.1111/adb.12224 25619621

[112] Shelly J Uhlmann A Sinclair H Howells FM Sibeko G Wilson D. First-Rank Symptoms in Methamphetamine Psychosis and Schizophrenia. Psychopathology (2016) 49:429–35. 10.1159/000452476 27926911

[113] Srisurapanont M Arunpongpaisal S Wada K Marsden J Ali R Kongsakon R. Comparisons of methamphetamine psychotic and schizophrenic symptoms: a differential item functioning analysis. Progr Neuro Psychopharmacol Biol Psychiatry (2011) 35:959–64. 10.1016/j.pnpbp.2011.01.014 21277930

[114] Tomiyama G. Chronic schizophrenia-like states in methamphetamine psychosis. Jpn J Psychiatry Neurol (1990) 44:531–9. 2074612 10.1111/j.1440-1819.1990.tb01626.x

[115] Wang LJ Lin SK Chen YC Huang MC Chen TT Ree SC. Differences in Clinical Features of Methamphetamine Users with Persistent Psychosis and Patients with Schizophrenia. Psychopathology (2016) 49:108–15. 10.1159/000445065 27071042

[116] Yamamuro K Makinodan M Kimoto S Kishimoto N Morimoto T Toritsuka M. Differential patterns of blood oxygenation in the prefrontal cortex between patients with methamphetamine-induced psychosis and schizophrenia. Sci Rep. (2015) 5:12107. 10.1038/srep12107 26178613 PMC4503985

[117] Hajebi A Amini H Kashani L Sharifi V. Twelve-month course and outcome of methamphetamine-induced psychosis compared with first episode primary psychotic disorders. Early Interv Psychiatry (2018) 12:928–34. 10.1111/eip.12404 27991722

[118] Ellinwood E Amphetamine psychosis: I. Description of the individuals and process. J Nerv Ment Dis. (1967) 144:273–83.

[119] Bousman CA McKetin R Burns R Woods SP Morgan EE Atkinson JH. Typologies of positive psychotic symptoms in methamphetamine dependence. Am J Addict. (2015) 24:94–7. 10.1111/ajad.12160 25864598 PMC4369467

[120] Fasihpour B Molavi S Shariat SV. Clinical features of inpatients with methamphetamine-induced psychosis. J Ment Health (2013) 22:341–9. 10.3109/09638237.2012.745184 23323572

[121] McKetin R Dawe S Burns RA Hides L Kavanagh DJ Teesson M. The profile of psychiatric symptoms exacerbated by methamphetamine use. Drug Alcohol Depend. (2016) 161:104–9. 10.1016/j.drugalcdep.2016.01.018 26874915

[122] Srisurapanont M Ali R Marsden J Sunga A Wada K Monteiro M. Psychotic symptoms in methamphetamine psychotic in-patients. Int J Neuropsychopharmacol. (2003) 6:347–52. 10.1017/S1461145703003675 14604449

[123] Yui K Ikemoto S Ishiguro T Goto K. Studies of amphetamine or methamphetamine psychosis in Japan: relation of methamphetamine psychosis to schizophrenia. Ann N Y Acad Sci. (2000) 914:1–12. 10.1111/j.1749-6632-2000.tb05178.x 11085303

[124] Angrist B Sathananthan G Wilk S Gershon S. Amphetamine psychosis: behavioral and biochemical aspects. J Psychiatr Res. (1974) 11:13–23. 4461784 10.1016/0022-3956(74)90064-8

[125] Bell D. Comparison of amphetamine psychosis and schizophrenia. Br J Psychiatry (1965) 111:701–7. 14337419 10.1192/bjp.111.477.701

[126] Dore G Sweeting M. Drug-induced psychosis associated with crystalline methamphetamine. Aus Psychiatry (2006) 14:86–9. 10.1111/j.1440-1665.2006.02252.x 16630206

[127] Yui K Ikemoto S Goto K. Factors for susceptibility to episode recurrence in spontaneous recurrence of methamphetamine psychosis. Ann N Y Acad Sci. (2002) 965:292–304. 10.1111/j.1749-6632.2002.tb04171.x 12105105

[128] Akiyama K. Longitudinal clinical course following pharmacological treatment of methamphetamine psychosis which persists after long-term abstinence. Ann N Y Acad Sci. (2006) 1074:125–34. 10.1196/annals.1369.012 17105910

[129] Mueser KT Bellack AS Brady EU. Hallucinations in schizophrenia. Acta Psychiatr Scand. (1990) 82:26–9. 2399817 10.1111/j.1600-0447.1990.tb01350.x

[130] Waters F Collerton D ffytche DH Jardri R Pins D Dudley R. Visual Hallucinations in the Psychosis Spectrum and Comparative Information From Neurodegenerative Disorders and Eye Disease. Schizophrenia Bull. (2014) 40:S233–45. 10.1093/schbul/sbu036 24936084 PMC4141306

[131] Rusyniak DE. Neurologic manifestations of chronic methamphetamine abuse. Psychiatric Clinics (2013) 36:261–75. 10.1016/j.psc.2013.02.005 23688691 PMC3764482

[132] Ali R Marsden J Srisurapanont M Sunga A Baigent M Monteiro M Methamphetamine Psychosis in Australia, Philippines, and Thailand: recommendations for acute care and clinical inpatient management. Addict Disord Their Treatm. (2010) 9:143–9. 10.1097/ADT.0b013e3181cf58f2

[133] Foussias G Agid O Fervaha G Remington G. Negative symptoms of schizophrenia: Clinical features, relevance to real world functioning and specificity versus other CNS disorders. Euro Neuropsychopharmacol. (2014) 24:693–709. 10.1016/j.euroneuro.2013.10.017 24275699

[134] Möller H-J. Clinical evaluation of negative symptoms in schizophrenia. Euro Psychiatry (2007) 22:380–6. 10.1016/j.eurpsy.2007.03.010 17524626

[135] Bobes J Arango C Garcia-Garcia M Rejas J. Healthy lifestyle habits and 10-year cardiovascular risk in schizophrenia spectrum disorders: An analysis of the impact of smoking tobacco in the CLAMORS schizophrenia cohort. Schizophrenia Res. (2010) 119:101–9. 10.1016/j.schres.2010.02.1030 20219322

[136] Makinen J Miettunen J Isohanni M Koponen H. Negative symptoms in schizophrenia—A review. Nordic J Psychiatry (2008) 62:334–41. 10.1080/08039480801959307 18752104

[137] Bushnell MC Goldberg ME Robinson DL. Behavioral enhancement of visual responses in monkey cerebral cortex. I Modulation in posterior parietal cortex related to selective visual attention. J Neurophysiol. (1981) 46:755–72. 10.1152/jn.1981.46.4.755 7288463

[138] Akiyama K Saito A Shimoda K. Chronic methamphetamine psychosis after long-term abstinence in Japanese incarcerated patients. Am J Addict. (2011) 20:240–9. 10.1111/j.1521-0391.2011.00124.x 21477052

[139] Mullen JM Crawford AT Amphetamine Related Psychiatric Disorders. Treasure Island, FL: StatPearls; StatPearls Publishing StatPearls Publishing LLC (2018). 29493990

[140] Farnia V Shakeri J Tatari F Juibari TA Yazdchi K Bajoghli H. Randomized controlled trial of aripiprazole versus risperidone for the treatment of amphetamine-induced psychosis. Am J Drug Alcohol Abuse (2014) 40:s10–15. 10.3109/00952990.2013.861843 24359506

